# The relationship between injection status, cocaine use, overdose, and drug-related behaviors among people who use opioids

**DOI:** 10.1016/j.dadr.2025.100398

**Published:** 2025-11-24

**Authors:** Carl A. Latkin, Lauren Dayton, Haley Bonneau, Melissa A. Davey-Rothwell, Grace Tian Yi, Kaori Suga, Oluwaseun Falade-Nwulia

**Affiliations:** a Department of Health, Behavior and Society, Bloomberg School of Public Health, Johns Hopkins University, Baltimore, USA; b Division of Infectious Diseases, School of Medicine, Johns Hopkins University, Baltimore, MD, USA

**Keywords:** Injection drug use, Opioids, Cocaine, Polysubstance use, Overdose, Harm reduction

## Abstract

**Background:**

The co-use of opioids and stimulants is associated with elevated health risks. This study examined patterns of injection drug use and cocaine use frequency among people who use opioids.

**Methods:**

Cross-sectional data were collected from community-recruited adults who use opioids in Baltimore, Maryland, between December 7, 2022 and January 26, 2025. Participants were categorized into four groups based on injection status and cocaine use frequency. Multinomial logistic regression examined factors associated with group membership, calculating relative risk ratios (RRR) and adjusted relative risk ratios (aRRR) with 95 % confidence intervals.

**Results:**

Of 777 participants (60.7 % male, 71.6 % Black, median age 52), 29.2 % reported past-month injection drug use. Daily/almost daily use was reported for heroin/fentanyl (79.5 %), crack cocaine (43.2 %), and powder cocaine (9.3 %). Four distinct drug use patterns emerged: not inject/lower frequency cocaine use (42.0 %), inject/lower frequency cocaine use (9.3 %), not inject/higher frequency cocaine use (28.8 %), and inject/higher frequency cocaine use (19.9 %). Factors significantly associated with injecting and higher frequency cocaine use group membership included: overdose history (aRRR=2.58, 95 % CI=1.52–4.38), withdrawal behavior with high overdose risk (aRRR=1.29, 95 % CI=1.14–1.46), and using in multiple locations (aRRR=1.09, 95 % CI=1.02–1.18).

**Conclusions:**

Nearly one-fifth of people who use opioids reported both injection drug use and high-frequency cocaine use, and greater frequency of cocaine use was associated with higher overdose risk. Targeted interventions addressing polysubstance use patterns, social networks, and environmental factors are urgently needed to reduce harm among this high-risk population.

## Highlights

• People who use opioids had four drug patterns by cocaine use frequency and injection status, each with distinct behaviors.
• High-frequency cocaine use was associated with increased recent overdose for both injectors and non-injectors.
• High-frequency cocaine use, both injecting and not, was associated with more overdose-risk behaviors and buying drugs closer to home.
• People who injected opioids were much more likely to be White than Black race.

## 1 Introduction

Stimulant and injection opioid use are both risk factors for fatal overdose and other adverse health outcomes among people who use opioids (Ciccarone and Shoptaw, 2022, Palis et al., 2022, Van Amsterdam and Van Den Brink, 2025). Much of the prior research on comparing the overdose risk of people who inject drugs (PWID) and those who consume opioids by other routes occurred before the introduction of fentanyl into the illicit opioid supply. Prior to the fentanyl epidemic, injection was the predominant route of administration for street-acquired opioids, particularly black tar heroin, which is less suitable for intranasal or smoking routes (Ciccarone, 2009). In North America, where white-powder heroin was most common, injection drug use was highly prevalent among people who use opioids (Ciccarone, 2009). However, there appears to have been a marked change in the routes of administration with fentanyl in the US. Based on the substance use treatment assessment data from 14 states comparing rates in 2017 to 2023, injecting fentanyl sharply declined across the U.S., with regional differences of snorting/sniffing increasing in the Northeast, and smoking increasing in the Midwest, South, and West regions (Chen et al., 2024).

Research before the fentanyl era suggested that injection opioid use is linked to severity of drug use disorder and individuals often transition to injection as their level of severity of drug use disorder increases (Carpenter et al., 1998, Mars et al., 2014, Neaigus et al., 2006, Novak and Kral, 2011). A systematic review and meta-analysis of cohort studies of opioid dependent individuals found that the percentage of injectors predicted mortality rates (Degenhardt et al., 2011). Studies have documented greater health consequences of injecting compared to other modes of drug use. These health consequences include bloodborne infections such as HIV, HCV, and endocarditis due to intravenous drug administration (Degenhardt et al., 2016, Larney et al., 2017, Levitt et al., 2020). Other consequences, such as increased risk of overdose, are likely due to pharmacokinetic factors, with injection offering a more rapid and efficient delivery of opioids, compared to other modes of administration (Cone et al., 1993).

There are also social factors that contribute to injection behaviors. Injectors often affiliate with others who inject, creating social networks that reinforce and normalize injection practices, which perpetuate injection drug use (Costenbader et al., 2006, De et al., 2007). However, with the rise of fentanyl, which is a highly potent synthetic opioid, there are now fewer people using opioids who administer their drugs by injection. With the staggering increase in overdose fatalities, there has been an expansion in the availability of medications for opioid use disorder (MOUD) (Volkow, 2021). The reduction in the proportion of opioids administered through injection and the increase in MOUD access are beneficial for public health. Yet, the growing role of stimulants, particularly cocaine, on the east coast of the US, in driving fatal overdoses remains unaddressed, since MOUD does not target stimulant use (Kariisa et al., 2019). This underscores the need for research to understand the co-use of opioids and stimulants.

The concurrent use of cocaine and opioids is a common practice (Palis et al., 2022). Cocaine, a central nervous system stimulant, can help to counteract some of the sedative properties of opioids. Studies of non-human animals find that the combination of cocaine plus heroin is more reinforcing than either drug alone. In addition, cocaine may ameliorate the severity of opioid withdrawal symptoms (Hansen et al., 2021, Maguire and Minervini, 2022). Strickland et al. (2022) suggest that for individuals who are physically dependent on opioids, dopamine supersensitivity follow abstinence may make stimulants highly reinforcing.

The widespread infiltration of fentanyl in the illicit drug supply has significant implications for drug use patterns and withdrawal management. Fentanyl can lead to greater withdrawal severity and faster onset compared to heroin due to its shorter half-life, increasing the frequency and intensity of withdrawal (Rook et al., 2006, Sharma et al., 2025). As a result, some individuals who use drugs may use cocaine to decrease the severity of withdrawal symptoms. However, this pattern of co-use heightens overdose risk due to the physiological effects of stimulants, including increasing heart rate and oxygen consumption, which can exacerbate the respiratory depression caused by opioids and contribute to fatal overdose (Maguire and Minervini, 2022).

Prior research suggests that the type and frequency of drug use are associated with social and demographic variables. For example, social network factors are associated with the type of drug use (Barman-Adhikari et al., 2015, Knerich et al., 2019). Individuals with greater severity of drug use disorder are more likely to use in high-risk settings such as shooting galleries and other public or semipublic locations (Werb et al., 2016). Demographic factors have also been linked to the type of drug use, with greater crack use among women and racial differences in drug use patterns and practices (Chilcoat and Schütz, 1995, Lejuez et al., 2007). In the current study we found women were more likely than men to report high frequency cocaine use and the form of cocaine was predominantly crack. Prior research suggests that gender differences in crack use may be due to economic marginalization and survival strategies, gendered relationships and power dynamics, and trauma (Sterk, 1999, Victor and Hedden-Clayton, 2023, Bourgois 2000).

Given the prior evidenceon the elevated risks of injecting opioids compared to other modes of administration, the dangers of the combined use of opioids and cocaine, and the prevalence of fentanyl in the drug supply, this study applied a social ecological model to examine social network, environmental, and individual drug use and overdose-risk profiles across four groups: opioid injectors versus non-injectors, and within each group, frequent versus infrequent or non-use of cocaine.

## 2 Methods

Study participants were enrolled in the OASIS study (Yi, et al., 2025). The study examined geospatial factors linked to drug overdoses and trained individuals to distribute overdose prevention and care materials. Participants were recruited in the community, targeting areas with high drug use in Baltimore, Maryland. Enrollment criteria included illicit opioid use in the prior 30 days, age of 18 years or older, and current residence in the Baltimore Metropolitan area. All study protocols were approved by the Bloomberg School of Public Health IRB. Participants received USD 40 for the study visits. From December 7, 2022 to January 26, 2025, 792 participants were recruited into the study.

To assess drug use, participants were first asked about the last time they used specific drugs (heroin, cocaine, crack, methamphetamine, buprenorphine, and fentanyl). Those who reported use in the past three months were asked about the frequency of use in the last three months. The question to assess fentanyl use was: “In the past 3 months, how often did you use fentanyl? This includes using fentanyl alone or other drugs mixed with fentanyl.” The response categories were: “Never,” “Less than monthly,” “Monthly,” “Weekly,” “Daily or Almost Daily.” The frequency of drug use categories was based on the European School Survey Project on Alcohol and Other Drugs 2019 survey and survey piloting, where we found that the category of daily or almost daily was more likely to be endorsed by respondents who were primarily used daily but could remember a brief period when they didn’t use for a day (European Monitoring Centre for Drugs and Drug Addiction., 2020). Frequencies of drug use for fentanyl and/or heroin, crack cocaine, and powder cocaine were then categorized into binary variables for high frequency use (daily or almost daily) compared to lower frequency use (less than daily or almost daily use). For the analyses, frequencies of crack and powder cocaine use were combined into high frequency (daily or almost daily) use of crack and/or cocaine compared to less than daily or almost daily use of crack and/or powder cocaine. We also did not include methamphetamine in the model, as frequent use was less than 2 %.

To assess injection drug use, participants were asked, “When was the last time that you injected drugs?” Response categories were: “More than 12 months ago,” “7–12 months ago,” “4–6 months ago,” “1–3 months ago,” “Within the past 4 weeks,” “Within the past 2 weeks,” “Within the past week,” “Within the past day,” and “Never”. Current injection drug use was defined as injecting in the prior four weeks. However, there were only 14 individuals (1.8 %) who reported injecting in the prior four weeks and not in the last two weeks, and analyses using the cutoff of the prior two weeks did not appreciably change the results.

Two variables examined drug social network factors. These variables included a binary yes/no variable in response to the question “Do you have a running buddy? (A running buddy is someone that you hustle with, go in together with, and/or often use with.)” The second question was: “How often do you buy drugs WITH the same people?”, with the response options of “Always,” “Often,” “Sometimes,” “Rarely,” “Never,” and “I only buy alone.”

The question, “In general, how far from where you live do you typically buy drugs?” assessed the distance traveled from residence to obtain drugs. The response categories were: “Same block,” “Near my block,” “In my neighborhood,” “Outside my neighborhood,” and collapsed for analyses into in the neighborhood and outside the neighborhood. The number of places where drugs were used was obtained by the question, “Thinking about the places that you use drugs, how many different places have you used in the past month (30 days)?” The responses were coded 1 to 10 and 11 or more.

Two questions assess drug overdose history. Participants were asked, “How many times in your life have you overdosed?” Recent overdose was assessed with the question, “When was your most recent overdose?” with the response options of “Within the past 6 months,” “6 months-1 year ago,” “Greater than 1 year ago,” and “Never.” As the focus was on a recent overdose, those who reported overdosing in the prior six months were compared to all others.

Since we were interested in how drug use patterns may increase risk behaviors associated with withdrawal symptoms, we developed a brief measure of withdrawal overdose risk behavior. The measure included the two questions, “Sometimes I care more about getting high than my own safety” and “Sometimes when I feel sick or in withdrawal from not having my fix, I don't think about overdose prevention.” The response options were “Strongly Agree,” “Agree,” “Neither Agree nor Disagree,” “Disagree,” and “Strongly Disagree.” These items were added together, resulting in a score range of 2–10, with a higher score indicating greater agreement.

Three questions determined MOUD drug treatment: “Have you ever been in drug treatment?”, “When were you most recently in drug treatment?” and “Are you currently taking any of the following medications to treat drug addiction: buprenorphine/Suboxone, methadone, Naltrexone, or some other medication?” An affirmative response to buprenorphine/suboxone, methadone, naltrexone, or some other medication was categorized as currently on MOUD.

Demographic questions included age, sex, race, education level, and homelessness. Race was self-reported, and the response categories for race were “African American/Black,” “White,” “Asian,” “Other,” and “Refuse to answer.” As there were few responses in the categories of “Asian,” “Other,” and “Refuse to answer,” these responses were collapsed into an “Other” category. Sex was assessed as “Male” and “Female” assigned at birth. The highest level of education that you have completed was categorized as “Grade 11 or less,” “Grade 12 or GED,” “Some college, Associate’s degree, or Technical degree,” and “Bachelor’s degree or greater.” Experiencing homelessness was assessed with the item, “In the past 6 months, have you slept for at least one week in the following places: a squatting place, an abandoned building, a car, a shelter, a park, or on the street?”

## 3 Analyses

The study survey was completed by 792 respondents. Analyses were restricted to respondents who reported illicit opioid use in the prior four weeks on both the screener and baseline survey, which led to the dropping of 11 cases, and four cases were removed due to missing data for a final analytic sample size of 777. Based on the hypothesized differences in patterns of drug use, the primary outcome variables were four mutually exclusive groups based on injection status and frequency of crack and/or powder cocaine use:

1. Did not inject and no daily or almost daily crack/cocaine use (Not Inject, Lower Frequency Cocaine Use)
2. Injected and no daily or almost daily crack/cocaine use (Inject, Lower Frequency Cocaine Use)
3. Did not inject and daily or almost daily crack/cocaine use (Not Inject, High Frequency Cocaine Use)
4. Injected and daily or almost daily crack/cocaine use (Inject, High Frequency Cocaine Use)

Descriptive statistics were calculated for all study variables. Multinomial logistic regression models were then used to examine associations with group membership using the “Not Inject, Lower Frequent Cocaine Use” as the reference group. All four outcomes were modeled simultaneously. Both unadjusted and adjusted relative risk ratios (aRRR) and 95 % confidence intervals (CI) were calculated. Stata 17 (College Station, TX: StataCorp LLC) was used for analyses. The adjusted multinomial regression models included the covariates from the unadjusted models with p< .15. However, all demographic variables were included in the adjusted multinomial regression models. Multicollinearity was assessed using VIF and all values were found to be less than 1.5.

## 4 Results

Of the 777 participants (Table 1), 60.7% were male and 39.3 % female. The median age was 52, the mean 49.8 (SD=10.9, range of 53 years (22-75), the 25th percentile was 41, and the 75th percentile was 58. The sample was predominantly Black (71.6 %), with 22.8 % White and 5.7 % identifying as “Other.” Educational attainment was low: 29.1 % had ≤  Grade 11, 45.3 % completed Grade 12 or GED, 22.9 % had some college or an associate’s/technical degree, and only 2.7 % held a bachelor’s degree or higher. Nearly half (42.2 %) reported experiencing homelessness for at least one week in the past six months.

**Table 1:** OASIS Study Participant Characteristics (N = 777).

**Variable**	**Category**	**Frequency (%)**				
		**Total**	Not inject, Lower frequency cocaine use	Inject, Lower Frequency Cocaine Use	Not Inject, High Frequency Cocaine Use	Inject, High Frequency Cocaine Use
Sex at birth	Male	472 (60.7)	216 (66.3)	49 (68.1)	113 (50.4)	94 (60.7)
	Female	305 (39.3)	110 (33.7)	23 (31.9)	111 (49.6)	61 (39.3)
Race	Black	556 (71.6)	285 (87.4)	33 (45.8)	174 (77.7)	64 (41.3)
	White	177 (22.8)	26 (8.0)	33 (45.8)	36 (16.1)	82 (52.9)
	Other	44 (5.7)	15 (4.6)	6 (8.3)	14 (6.2)	9 (5.8)
Education level	Grade 11 or less	226 (29.1)	100 (30.7)	18 (25.0)	72 (32.1)	36 (23.2)
	Grade 12 or GED	352 (45.3)	140 (42.9)	41 (56.9)	95 (42.4)	76 (49.0)
	Some college, Associate’s, Technical degree	178 (22.9)	78 (23.9)	10 (13.9)	49 (21.9)	41 (26.5)
	Bachelor’s degree or greater	21 (2.7)	8 (2.5)	3 (4.2)	8 (3.6)	2 (1.3)
Homeless in past 6 months	Yes	328 (42.2)	87 (26.7)	37 (51.4)	95 (42.4)	109 (70.3)
Daily or almost daily drug use in the past 3 months*	Heroin and or fentanyl	618 (79.5)	233 (71.5)	56 (77.8)	187 (83.5)	142 (91.6)
	Fentanyl	517 (76.3)	175 (53.7)	51 (70.8)	159 (71.0)	132 (85.2)
	Heroin	534 (68.7)	206 (63.2)	47 (65.3)	157 (70.1)	124 (80.0)
	Crack	336 (43.2)	0 (0.0)	0 (0.0)	205 (91.5)	131 (84.5)
	Powdered cocaine	72 (9.3)	0 (0.0)	0 (0.0)	29 (13.0)	43 (27.7)
	Prescription opiates to get high	66 (8.5)	32 (9.8)	4 (5.6)	11 (4.9)	19 (12.3)
	Buprenorphine to get high	18 (2.3)	9 (2.8)	1 (1.4)	3 (1.3)	5 (3.23)
	Methamphetamine	11 (1.4)	4 (1.2)	0 (0.0)	2 (1.0)	5 (3.2)
Injection drug use in the past month	Yes	227 (29.2)	0 (0.0)	72 (100.0)	0 (0.0)	155 (100.0)
Currently on medication for opioid use disorder	Yes	382 (49.2)	160 (49.1)	46 (63.9)	102 (45.5)	74 (47.7)
Overdose in the past 6 months	Yes	183 (23.6)	54 (16.6)	17 (23.6)	62 (27.7)	50 (32.3)
Distance from home to where drugs are typically purchased	Same block	92 (11.8)	24 (7.4)	6 (8.3)	33 (14.7)	29 (18.7)
	Nearby block	142 (18.3)	45 (13.8)	14 (19.4)	47 (21.0)	36 (23.2)
	In neighborhood	222 (28.6)	93 (28.5)	24 (33.3)	61 (27.2)	44 (28.4)
	Outside of neighborhood	321 (41.3)	164 (50.3)	28 (38.89)	83 (37.1)	46 (20.7)
How often buy drugs with the same people	Always	207 (26.6)	83 (25.5)	21 (29.2)	67 (29.9)	36 (23.2)
	Often	297 (38.2)	111 (34.1)	23 (31.9)	90 (40.2)	73 (47.1)
	Sometimes	146 (18.8)	73 (22.4)	14 (19.4)	29 (13.0)	30 (19.4)
	Rarely	62 (8.0)	22 (6.8)	9 (12.5)	21 (9.4)	10 (6.5)
	Never	20 (2.6)	10 (3.1)	2 (2.8)	7 (3.1)	1 (0.7)
	Only buy alone	45 (5.8)	27 (8.3)	3 (4.2)	10 (4.5)	5 (3.2)
Have a running buddy	Yes	474 (61.0)	183 (56.1)	53 (73.6)	133 (59.4)	105 (67.7)
Withdrawal risk behaviors scale (mean, SD)		6.94, 2.14	6.34, 2.18	6.94, 2.16	7.17, 2.03	7.88, 1.82
Number of places where drugs were used (mean, SD)		4.79, 3.47	3.82, 3.10	5.64, 3.53	4.68, 3.34	6.57, 3.61
Injection drug use status and frequency of cocaine use	Not inject, Lower frequency cocaine use	326 (42.0)	---	---	---	---
	Inject, Lower frequency cocaine use	72 (9.3)	---	---	---	---
	Not inject, High frequency cocaine use	224 (28.8)	---	---	---	---
	Inject, High-frequency cocaine use	156 (19.9)	---	---	---	---

⁎ - Among those who reported that they had used the drug in the prior three months.

Almost half (49.2 %) reported that they were currently receiving medication for opioid use disorder (Table 1). Nearly one in four (23.6 %) experienced at least one overdose in the past six months. Regarding drug purchasing behavior, 41.3 % traveled “outside of neighborhood” to buy drugs, 28.6 % within their neighborhood, and only 11.8 % on their own block; most purchased with the same people “often” (38.2 %) or “always” (26.6 %). Sixty-one percent reported having a “running buddy.” On average, participants scored 6.94 (SD=2.14) from a score range of 2–10 on the withdrawal overdose risk behavior scale and used drugs in 4.79 (SD=3.47) distinct locations. There was a high level of cocaine and opioid use in the prior three months: 30.7 % powder cocaine, 68.0 % crack, 23.3 % prescription opiates to get high, 10.8 % buprenorphine to get high, 7.9 % methamphetamine, 87.1 % fentanyl, and 86.9 % heroin. One third of participants (32.3 %) reported injection drug use in the past 3 months. Substance-use patterns show, among participants who reported use of these drugs in the past three months, (Table 1) that 79.5 % used heroin or fentanyl daily or almost daily over the past three months, and 43.2 % used crack daily or almost daily; by contrast, 9.3 % used powder cocaine, and 1.4 % used methamphetamine at that frequency.

Regarding cocaine use, 60.5 % reported crack use in the prior week, while only 19.8 % reported using powder cocaine in the prior week. Opioid use in the prior week was highly prevalent; 82.2 % reported heroin use, and 80.1 % reported fentanyl use. Respondents who reported cocaine, crack, heroin, and fentanyl use in the prior three months were asked an additional question about the frequency of use in the prior three months. Among those who used crack, 43.2 % reported daily or almost daily use. Most (68.7 %) participants reported daily or almost daily heroin use, and for fentanyl use, it was 76.3 %.

Slightly less than a third of the sample (29.2 %) reported injection drug use in the prior month. Among the injectors, 57.7 % reported high frequency crack use and 18.9 % reported high frequency powder cocaine use. Whereas among the non-injectors, 37.3 % reported high-frequency crack use and only 5.3 % reported high-frequency powder cocaine use.

The multivariable multinomial regression models compared three groups to those in the reference group who did not inject and did not report frequent use of cocaine (Table 2). The largest group was not inject/lower frequency cocaine use (42.0 %), followed by not inject/higher frequency cocaine use (28.8 %), inject/higher frequency cocaine use (19.9 %), and inject/lower frequency cocaine use (9.3 %).

**Table 2:** Multivariate Multinomial Logistic Regression of injection status and cocaine frequency with the comparison group of not inject, lower frequency cocaine use. (n = 777).

	Model 1: Inject, Lower Frequency Cocaine Use		Model 2: Not Inject, High Frequency Cocaine Use		Model 3: Inject, High Frequency Cocaine Use	
	RRR (95 % CI)	aRRR (95 % CI)	RRR (95 % CI)	aRRR (95 % CI)	RRR (95 % CI)	aRRR (95 % CI)
Education (Grade 12/GED or more vs less)	1.33 (0.74,2.38)	1.15 (0.61,2.16)	0.93 (0.65,1.35)	1.04 (0.70,1.55)	1.46 (0.94,2.27)	1.66 (0.98,2.83)
Sex (Female vs Male)	0.92 (0.53,1.59)	0.80 (0.43,1.47)	**1.93** **(1.36,2.73)**	**2.02** **(1.38,2.94)**	1.27 (0.86,1.89)	1.41 (0.87,2.31)
Age	**0.95** **(0.93,0.98)**	0.99 (0.96,1.02)	**0.98** **(0.96,1.00)**	1.00 (0.98,1.02)	**0.93** **(0.91,0.95)**	**0.98** **(0.95,1.00)**
Race: White vs Black	**10.96** **(5.85,20.54)**	**8.30** **(3.98,17.31)**	**2.27** **(1.32,3.89)**	1.49 (0.82,2.73)	**14.04** **(8.37,23.57)**	**6.35** **(3.40,11.86)**
Race: Other vs Black	**3.46** **(1.25,9.51)**	**3.72** **(1.26,11.05)**	1.53 (0.72,3.24)	1.21 (0.54,2.72)	**2.67** **(1.12,6.38)**	1.58 (0.57,4.39)
Homelessness (past 6 mo)	**2.90** **(1.72,4.90)**	1.35 (0.73,2.51)	**6.51** **(4.26,9.94)**	1.48 (0.97,2.27)	**2.90** **(1.72,4.90)**	**2.56** **(1.52,4.32)**
Current MOUD use	**1.84** **(1.08,3.11**)	1.75 (0.99,3.09)	0.87 (0.62,1.22)	0.84 (0.59,1.22)	0.95 (0.65,1.39)	0.97 (0.60,1.55)
Daily or almost daily heroin/fentanyl use (past 3 mo)	1.40 (0.76,2.56)	1.36 (0.69,2.69)	**2.02** **(1.32,3.09)**	**2.02** **(1.28,3.18)**	**4.36** **(2.35,8.08)**	**4.24** **(2.07,8.69)**
Distance to purchase (> neighborhood vs ≤ neighborhood)	0.64 (0.35,1.16)	0.72 (0.41,1.26)	**0.59** **(0.40,0.88)**	**0.64** **(0.44,0.93)**	**0.24** **(0.13,0.43)**	**0.54** **(0.33,0.87)**
Overdose (past 6 mo)	1.56 (0.84,2.89)	1.57 (0.80,3.06)	**1.93** **(1.27,2.92)**	**1.75** **(1.13,2.70)**	**2.40** **(1.54,3.75)**	**2.58** **(1.52,4.38)**
Buying with the same people	1.06 (0.86,1.31)	0.87 (0.67,1.13)	**1.15** **(1.00,1.33)**	1.10 (0.93,1.29)	**1.19** **(1.00,1.40)**	0.99 (0.79,1.23)
Running buddy	**2.18** **(1.24,3.85)**	**2.19** **(1.11,4.33)**	1.14 (0.81,1.61)	0.86 (0.57,1.29)	**1.64** **(1.10,2.45)**	1.09 (0.64,1.87)
Withdrawal risk	**1.32** **(1.08,1.61)**	1.02 (0.89,1.17)	**1.34** **(1.18,1.54)**	**1.15** **(1.05,1.26)**	**1.85** **(1.57,2.19)**	**1.29** **(1.14,1.46)**
Number of places where use drugs	**1.18** **(1.09,1.27)**	1.08 (0.99,1.18)	**1.09** **(1.03,1.15)**	1.05 (0.98,1.12)	**1.26** **(1.19,1.33)**	**1.09** **(1.02,1.18)**

RRR- unadjusted relative risk ratio, aRRR- adjusted relative risk ratio, 95 % CI- 95 % confidence interval.

Bold- statistically significant at p < .05

Compared to the reference group (not injecting, lower frequency cocaine use), in the multivariable analyses, the Inject, Lower-Frequency Cocaine Use Group revealed the following association: significantly more likely to be White, compared with Black participants (aRRR=8.30, 95 % CI=3.98–17.31). Members of this group were also more likely to be of “Other” races versus Blacks (aRRR=3.72, 95 % CI=1.26–11.05). Having a running buddy doubled the adjusted risk of being in the inject lower-frequency cocaine use group (aRRR=2.19, 95 % CI=1.11–4.33). All other variables were not statistically significant in this comparison after adjustment. However, age, withdrawal risk behavior, number of places used, current MOUD, and having a running buddy were statistically significant in the bivariate models but not in the adjusted models.

Not Inject, High-Frequency Cocaine Use: Females had about twice the adjusted risk of membership in the not inject, high-frequency cocaine use group (aRRR=2.02, 95 % CI=1.38–2.94) compared to the not inject, lower-frequency cocaine use group. Participants reporting daily heroin/fentanyl use were also roughly twice as likely to belong to the high-frequency cocaine, non-injecting group (aRRR=2.02, 95 % CI=1.28–3.18). Individuals with a recent overdose had a 75 % greater adjusted relative risk of belonging to the non-injecting, high-frequency cocaine use group (aRRR = 1.75, 95 % CI = 1.13–2.70). Traveling outside one’s neighborhood to buy drugs was negatively associated with not inject, high-frequency cocaine use group (aRRR=0.64, 95 % CI=0.44–0.93). Each one-point increase in withdrawal risk behavior score raised the adjusted risk for being in the not inject, high-frequency cocaine use group by 15 % (aRRR=1.15, 95 % CI=1.05–1.26). Although White race, homelessness, buying with the same people, and number of places used drugs were associated with not inject, high-frequency cocaine use group membership in the unadjusted models, they did not retain statistical significance in the adjusted model. Other covariates—race, running buddy, current MOUD use, buying with the same people, number of places used, age, and education were not significant in the adjusted models.

Inject, High-Frequency Cocaine Use: Compared to the not inject, lower frequency cocaine use group, Whites were over six times as likely as Blacks to be in the inject and use cocaine at high frequency group (aRRR=6.35, 95 % CI=3.40–11.86). Age was negatively associated with injecting and high-frequency cocaine use group membership (aRRR=0.98, 95 % CI=0.95–1.00). Those in the inject, high-frequency cocaine group had a four-fold higher adjusted risk of high-frequency heroin/fentanyl use (aRRR=4.24, 95 % CI=2.07–8.69). A recent overdose was associated with a 158 % higher adjusted risk of membership in the injecting, high-frequency cocaine use category (aRRR=2.58, 95 % CI=1.52–4.38). Participants experiencing homelessness in the prior six months had 2.56 times greater adjusted risk of inject, high-frequency cocaine use group membership (aRRR=2.56, 95 % CI=1.52–4.32). A one-unit increase in withdrawal risk behavior score was associated with 29 % higher adjusted risk (aRRR=1.29, 95 % CI=1.14–1.46). Each additional location where drugs were used increased adjusted risk by 9 % (aRRR=1.09, 95 % CI=1.02–1.18). Purchasing outside one’s neighborhood reduced adjusted risk by 46 % (aRRR=0.54, 95 % CI=0.33–0.87). Other races, homelessness, buying with the same people, having a running buddy, and the number of places used were associated with inject, high-frequency cocaine use group membership in the unadjusted models; they did not retain statistical significance in the adjusted model.

## 5 Discussion

Several distinct patterns were observed in these data. Notably, unlike findings before the fentanyl epidemic, people who injected but did not frequently use cocaine were no more likely to use opioids daily/almost daily compared to non-injectors with similarly low cocaine use (Novak and Kral, 2011). Among people who did not frequently use cocaine, injectors were similar to non-injectors, with the exception of race. White participants were significantly more likely to report current injection drug use (aRRR=8.3) and having a running buddy (aRRR=2.2). These findings align with prior findings that White individuals are more likely to inject drugs compared to Black individuals (Broz and Ouellet, 2008). Additionally, the findings support previous research that close social ties, such as a “running buddy,” may be linked to drug use behaviors (Latkin et al., 2003). The lack of differences between injectors and non-injectors in these analyses may be partly due to the properties of fentanyl, which is highly prevalent in Maryland (Maryland Department of Health Behavioral Health Administration, 2025). Specifically, there may be a smaller perceived difference in dose effect by route of administration compared to heroin, making injection less necessary for achieving the desired effect. Given the health risks associated with injecting, especially given the new adulterants in the drug supply, encouraging changes in the route of administration remains a critical harm reduction approach (Kral et al., 2021).

In the multinomial multivariate models, individuals who frequently used cocaine showed similar patterns regardless of injection status as compared to those who reported lower cocaine use and did not inject. The primary differences were that, compared to the reference group, the participants who reported higher frequency cocaine use and did not inject were more likely to be female. In contrast, injectors who were frequently used cocaine were more likely to use drugs in multiple settings, travel shorter distances to obtain drugs, and be White. People with high-frequency cocaine user, regardless of injection status, were also more likely to use heroin/fentanyl frequently, consistent with longstanding patterns of polysubstance use. This may be an indication of higher severity of drug use disorder and mutually reinforcing attributes.

High-frequency cocaine use, both among injectors and non-injectors, was strongly associated with higher scores on the brief withdrawal risk behavior scale, suggesting that individuals may be using cocaine, in part, to alleviate opioid withdrawal. Cocaine injection has been found to increase injection risk behaviors and HIV infection (Ciccarone and Shoptaw, 2022). However, it is not clear if the primary driver of risk is through cocaine use to reduce withdrawal symptoms or if frequent cocaine use leads to an additional risk path or moderates risk behavior, such as frequency of opioid use and use in higher risk settings. High-frequency cocaine use, regardless of injection status, was also associated with a lower likelihood of traveling outside one’s neighborhood to purchase drugs, though the reason remains unclear. Potential explanations include local drug availability, mobility constraints, or the need to stay close to income sources.

The study findings suggest that individuals who use both opioids and cocaine, most often in the form of crack, are at higher risk of a drug overdose and more frequent opioid use. The increased potency and shorter half-life of fentanyl compared to heroin may lead to greater cocaine use and related risk behaviors (Simpson et al., 2024). It is also possible that cocaine is used to counteract fentanyl’s sedative effects or to avoid crashing, which in turn creates a feedback loop of escalating polysubstance use. Fentanyl presents a double risk: its own overdose risk and its potential to reinforce high cocaine use.

Currently, there is no FDA-approved pharmacological treatment for cocaine use, and individuals who frequently use both cocaine and opioids remain challenging to treat. Contingency management has demonstrated efficacy in treating cocaine use disorder

(Lussier et al., 2006, Schierenberg et al., 2012). But additional strategies are urgently needed to address combined opioid and cocaine use. Reducing fentanyl use may help alleviate withdrawal symptoms and reduce the compensatory use of cocaine. However, both contextual factors (e.g., social network factors) and physiological factors (e.g., cravings) may impede efforts to reduce cocaine use.

In the current sample, MOUD was not associated in the multivariable models with high-frequency cocaine use, suggesting that individuals taking MOUD who continue to use opioids remain at risk for high-frequency cocaine use, particularly crack. Prior research suggests that insufficient MOUD dosage may impede effective treatment of fentanyl dependence (Bolshakova et al., 2024, Chambers et al., 2023, Cook et al., 2021). However, other social and structural factors should be considered, such as financial access to treatment, childcare, transportation, mental health services, and homelessness, which also serve as barriers to MOUD enrollment and retention (Farhoudian et al., 2022, Mojtabai et al., 2014). Due to the study design, which focused on people who were actively using opioids, we cannot determine the proportion of MOUD participants who are using illicit opioids or cocaine, nor do we have data on MOUD dosage.

The majority of cocaine use was in the form of crack, likely due to price and availability. Powder cocaine use was more closely associated with injection. Still, among people with high-frequency cocaine use and who injected, less than half (44.6 %) reported using powder cocaine in the prior week. While research on crack cocaine and health-related outcomes is mixed, substantial evidence links crack cocaine use with increased risk for STIs, HIV, and HCV (Butler et al., 2017). Future research should examine how fentanyl influences patterns of cocaine use, and whether factors such as cravings and using cocaine to cope with stress contribute to polysubstance use.

Study limitations should be noted. This sample was recruited based on opioid use and did not recruit individuals who reported that they were only using cocaine. The binary categorization of higher and lower frequency of cocaine use may have led to misclassifications; for example, people who use weekly may substantially differ from people who use monthly. While dichotomizing was intended to be conservative, some misclassification may remain due to self-report biases. We included the “almost daily” wording to reduce social desirability bias of under-reporting drug use. However, future studies could include daily recall (e.g., use on the prior day) to enhance precision. We also do not know about the actual drug content of the drugs consumed. They were likely to have psychoactive adulterants. The cross-sectional study design limits the ability to make causal inferences. We do not have information on the route of administration for the powder cocaine. Moreover, we did not assess migration patterns to and from Baltimore. Some of the racial differences may be due in part to White individuals moving into Baltimore from surrounding areas due to greater drug availability. Additional limitations include the lack of differentiation between crack and powder cocaine and between heroin and fentanyl in the analyses, reliance on self-reported measures, and potential Type I error inflation resulting from multiple comparisons.

## 6 Conclusion

The findings from this study suggest that among people who use opioids, those with lower-frequency cocaine use had fewer differences by injection status, aside from a higher likelihood of being White and reporting a “running buddy” among injectors. In contrast, high-frequency cocaine use was associated with elevated risk regardless of injection status. These risks included recent overdose, more frequent illicit opioid use, homelessness, and greater perceived risk during withdrawal. The persistence of these associations across both injectors and non-injectors underscores the urgent need for targeted interventions to address co-occurring opioid and cocaine use, particularly in the context of fentanyl dominated drug markets.

## CRediT authorship contribution statement

**Haley Bonneau:** Writing – review & editing, Formal analysis. **Melissa A. Davey-Rothwell:** Writing – review & editing, Supervision, Methodology. **Latkin Carl A:** Writing – review & editing, Writing – original draft, Project administration, Methodology, Funding acquisition, Formal analysis, Conceptualization. **Lauren Dayton:** Writing – review & editing, Project administration, Methodology, Investigation, Conceptualization. **Grace Tian Yi:** Writing – review & editing. **Kaori Suga:** Writing – review & editing. **Oluwaseun Falade-Nwulia:** Writing – review & editing.

## Financial disclosures

This work was supported in part by 10.13039/100000026NIDA under grants R01DA058659 and R01DA05047 and a grant from The Bloomberg American Health Initiative.

## Declaration of Competing Interest

The authors declare that they have no known competing financial interests or personal relationships that could have appeared to influence the work reported in this paper.
